# The new useful high-resolution computed tomography finding for diagnosing fibrotic hypersensitivity pneumonitis: “hexagonal pattern”: a single-center retrospective study

**DOI:** 10.1186/s12890-022-01869-4

**Published:** 2022-03-04

**Authors:** Hiroko Okabayashi, Taiki Fukuda, Tae Iwasawa, Tsuneyuki Oda, Hideya Kitamura, Tomohisa Baba, Tamiko Takemura, Takuro Sakagami, Takashi Ogura

**Affiliations:** 1 Department of Respiratory Medicine, Kanagawa Cardiovascular and Respiratory Center, 6-16-1 Tomioka-Higashi, Kanazawa-ku, Yokohama City, Kanagawa 236-0051 Japan; 2 Department of Radiology, Kanagawa Cardiovascular and Respiratory Center, 6-16-1 Tomioka-Higashi, Kanazawa-ku, Yokohama City, Kanagawa 236-0051 Japan; 3 Department of Pathology, Kanagawa Cardiovascular and Respiratory Center, 6-16-1 Tomioka-Higashi, Kanazawa-ku, Yokohama City, Kanagawa 236-0051 Japan; 4 Department of Respiratory Medicine, Kumamoto University Hospital, Faculty of Life Sciences, Kumamoto University, 1-1-1 Honjo, Chuo-ku, Kumamoto City, Kumamoto 860-8556 Japan; 5 Department of Radiology, The Jikei University School of Medicine, 3-19-18 Nishishinbashi, Minato-ku, Tokyo, 105-8471 Japan

**Keywords:** Fibrotic hypersensitivity pneumonitis, Idiopathic pulmonary pneumonitis, HRCT, Interlobular septal thickening, Hexagonal pattern

## Abstract

**Background:**

Centrilobular nodules, ground-glass opacity (GGO), mosaic attenuation, air trapping, and three-density pattern were reported as high-resolution computed tomography (HRCT) findings characteristic of fibrotic hypersensitivity pneumonitis (HP). However, it is often difficult to differentiate fibrotic HP from idiopathic pulmonary fibrosis (IPF). In fibrotic HP, the HRCT sometimes shows tortoiseshell-like interlobular septal thickening that extends from the subpleural lesion to the inner layers. This finding is called “hexagonal pattern,” and this study is focused on the possibility that such finding is useful for differentiating fibrotic HP from IPF.

**Methods:**

This study included patients with multidisciplinary discussion (MDD) diagnosis of fibrotic HP or IPF undergoing surgical lung biopsy between January 2015 and December 2017 in Kanagawa Cardiovascular and Respiratory Center. Two radiologists have evaluated the HRCT findings without clinical and pathological information.

**Results:**

A total of 23 patients were diagnosed with fibrotic HP by MDD and 48 with IPF. Extensive GGO, centrilobular nodules, and hexagonal pattern were more frequent findings in fibrotic HP than in IPF. No significant difference was observed between the two groups in the presence or absence of mosaic attenuation, air trapping, or three-density pattern. In the multivariate logistic regression, the presence of extensive GGO and hexagonal pattern was associated with increased odds ratio of fibrotic HP. The sensitivity and specificity of the diagnosis of fibrotic HP in the presence of the hexagonal pattern were 69.6% and 87.5%, respectively.

**Conclusion:**

Hexagonal pattern is a useful finding for differentiating fibrotic HP from IPF.

## Background

Two guidelines have recently been proposed for diagnosing hypersensitivity pneumonitis (HP), which state that lung biopsy is not necessary for the diagnosis of HP if antigen exposure, typical high-resolution computed tomography (HRCT) findings, and bronchoalveolar lavage (BAL) lymphocytosis have been identified [1, 2]. However, in fibrotic HP, the antigen is often unknown, and most cases need lung biopsy. In addition, specimens collected via transbronchial forceps biopsies or transbronchial lung cryobiopsy (TBLC) are insufficient in size for the diagnosis of fibrotic HP, and surgical lung biopsy (SLB) is often required. Conversely, SLB is often not feasible due to the patient’s background or facility issues. Therefore, HRCT imaging plays a significant role in the diagnosis of fibrotic HP.

HRCT findings characteristic of fibrotic HP include centrilobular nodules, ground-glass opacity (GGO), mosaic attenuation, air trapping, and three-density pattern in previous reports [1–8]. These findings are not specific to fibrotic HP only, and many cases of fibrotic HP were found without typical HRCT findings.

Approximately half of chronic HP has been reported to have histological usual interstitial pneumonia (UIP)-like pattern and extensive fibrosis with poor prognosis [9]. Fibrotic HP with UIP pattern is common and often difficult to distinguish from idiopathic pulmonary fibrosis (IPF) in clinical practice. However, differentiating between fibrotic HP and IPF is important as they differ in their treatment strategies.

**Fig. 1:**
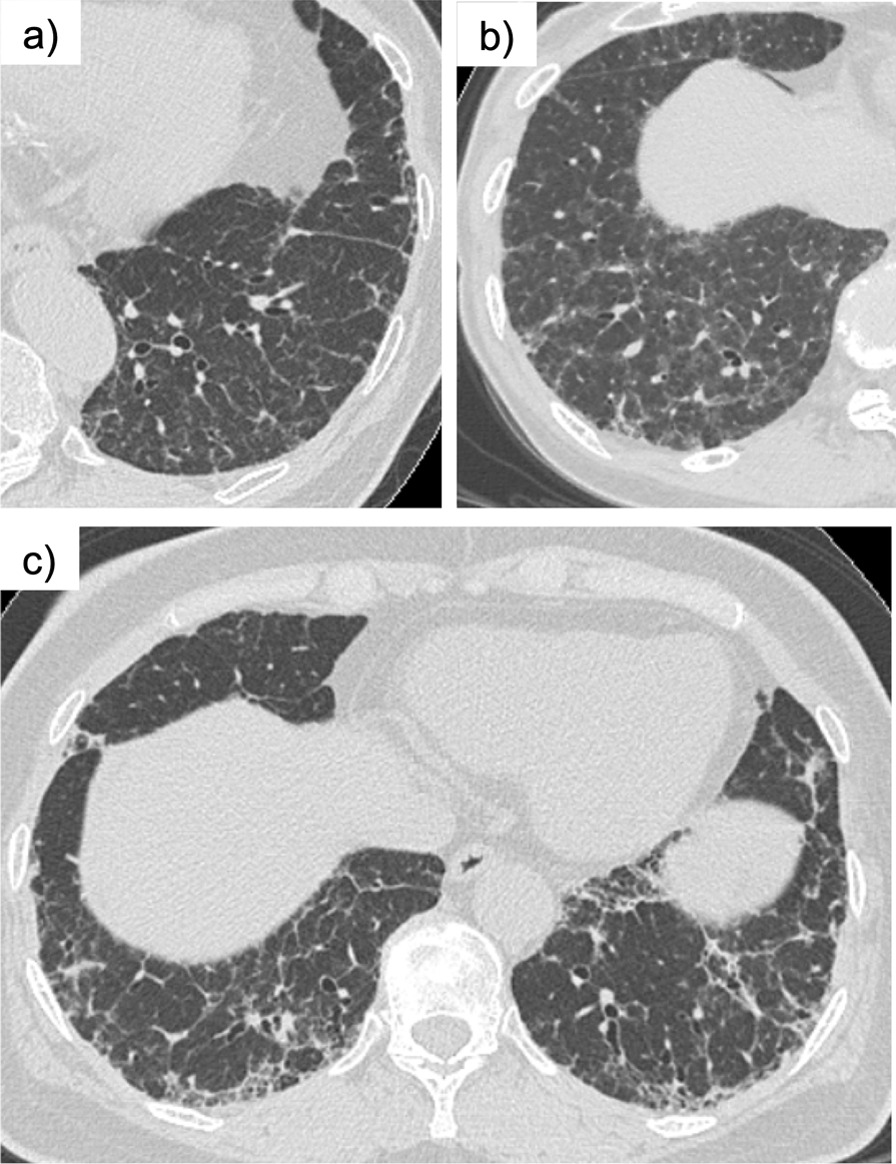
Hexagonal pattern in the HRCT. **a**, **b** Hexagonal pattern is an interlobular septal thickening expanding from the subpleural region to two or more inner layers of the secondary pulmonary lobules. **c** When there is subpleural collapse such that the structure of the secondary pulmonary lobules is no longer recognizable, hexagonal pattern was defined as interlobular septal thickening that expands to two or more inner layers of the secondary pulmonary lobules from the collapsed area

In fibrotic HP, the HRCT sometimes shows tortoiseshell-like interlobular septal thickening that extends from the subpleural region to the inner layers. This finding is called “hexagonal pattern,” and this study focused on the possibility that such finding is useful for differentiating fibrotic HP from IPF (Fig. 1).

## Methods

### Patients

This study included patients with multidisciplinary discussion (MDD) diagnosis of fibrotic HP or IPF undergoing SLB between January 2015 and December 2017 in Kanagawa Cardiovascular and Respiratory Center. The diagnosis of IPF and fibrotic HP was based on consensus using previously reported criteria [5, 10–12]. The exclusion criteria of this study were as follows: (1) patients that had collagen vascular diseases or pulmonary alveolar proteinosis, (2) patients that met the interstitial pneumonia with autoimmune features diagnostic criteria [13], (3) patients that developed collagen vascular disease following MDD, and (4) patients that have already been given medication, including steroids or immunosuppressant, before biopsy.

The patient’s baseline characteristics, including age, gender, smoking status, peripheral blood counts, blood biochemistry, pulmonary function tests, and BAL findings at the time of MDD, were retrospectively obtained from the medical records. This study was performed in accordance with the Declaration of Helsinki and approved by the Institutional Review Board of Kanagawa Cardiovascular and Respiratory Center (permission number: KCRC-20-0023, permission date: August 18, 2020). The opt-out consent was adopted for patient enrollment in this study.

### The definition of hexagonal pattern

Hexagonal pattern is defined as an interlobular septal thickening expanding from the subpleural region to two or more inner layers of secondary pulmonary lobules (Fig. 1a, b). When there is subpleural collapse such that the structure of secondary pulmonary lobules is no longer recognizable, hexagonal pattern was defined as interlobular septal thickening that expands to two or more inner layers of secondary pulmonary lobules from the collapsed area (Fig. 1c).

**Fig. 2:**
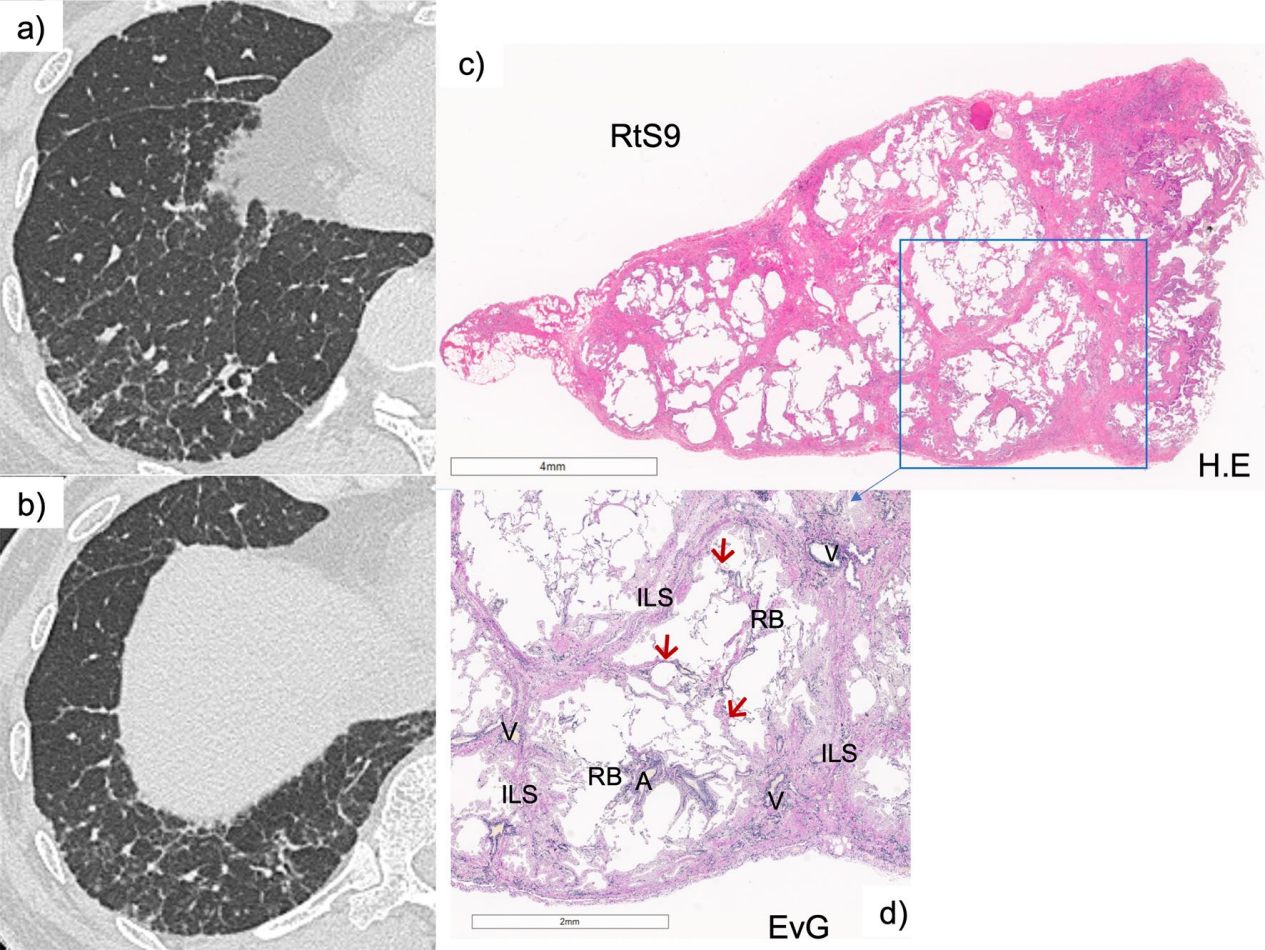
The pathological specimen of the patient with hexagonal pattern in HRCT. **a**, **b** The HRCT shows interlobular septal thickening expanding from the subpleural region to two or more inner layers of the secondary pulmonary lobules. **c**, **d** The specimen of right S9 obtained via surgical lung biopsy. Perilobular fibrosis and bridging fibrosis connecting the fibrosis in the centrilobular area and the fibrosis in the perilobular area were observed. The arrows indicate bridging fibrosis. In this case, the hexagonal pattern observed in the HRCT corresponded to perilobular fibrosis. ILS, interlobular septum; V, vein; A, artery; RB, respiratory bronchiole; HE, hematoxylin and eosin; EvG, Elastica van Gieson

Figure 2 presents the pathological specimen obtained via SLB of a patient with hexagonal pattern. Perilobular fibrosis and bridging fibrosis connecting the fibrosis in the centrilobular area and the fibrosis in the perilobular area were observed in this specimen. In this case, the hexagonal pattern observed in the HRCT corresponded to that of perilobular fibrosis.

### Radiological evaluation

The radiological data were reviewed by two chest radiologists (T.I. and D.F.). HRCT images were independently evaluated, and the final findings were agreed upon by consensus between the two radiologists. The radiologists knew that only patients with fibrotic HP or IPF were included in the study, but they evaluated the HRCT findings without clinical and pathological information. The two radiologists have retrospectively evaluated the shadow distribution and the following findings: hexagonal pattern, extensive GGO, centrilobular nodules, mosaic attenuation, air trapping, three-density pattern, reticulation, traction bronchiectasis, honeycombing, consolidation, emphysema, cyst, and pleuroparenchymal fibroelastosis. The HRCT findings were interpreted based on the recommendations of the Nomenclature Committee of the Fleischner Society [14]. In this study, mosaic attenuation, air trapping, and three-density pattern were defined as being present when they were found in three or more lobules bilaterally. Before the radiological evaluation, two teacher images were shown to confirm the definition of hexagonal pattern. The classification of cases as UIP, probable UIP, indeterminate for UIP, or alternative diagnosis using the American Thoracic Society (ATS)/European Respiratory Society (ERS)/Japanese Respiratory Society (JRS)/Latin American Thoracic Association (ALAT) guidelines for diagnosing IPF was performed retrospectively [12].

### Statistical analysis

Continuous variables are expressed as median values. The Mann–Whitney *U* test, chi-squared test, or Fisher’s exact probability test was employed, as appropriate, for between-group comparisons. The interobserver variation between two radiologists for the diagnosis of fibrotic HP or IPF was analyzed using κ statistic. A κ coefficient equal to or less than 0.20 indicated poor agreement; 0.21–0.40, fair agreement; 0.41–0.60, moderate agreement; 0.61–0.80, good agreement; and 0.81–1.00, excellent agreement. Univariate and multivariate logistic analyses were employed to identify the useful HRCT findings for distinguishing fibrotic HP from IPF. A *p*-value of < 0.05 was considered to be significant. All statistical analyses have been conducted using the Statistical Package for the Social Sciences software version 24.0 (IBM; Armonk, NY, USA).

## Results

### Patient characteristics

**Fig. 3:**
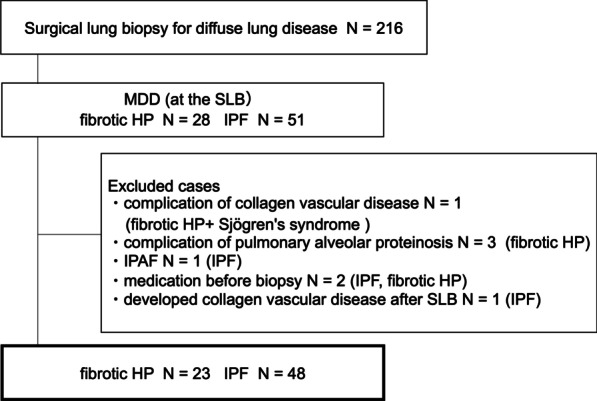
Patient flow diagram. MDD, multidisciplinary discussion; SLB, surgical lung biopsy; HP, hypersensitivity pneumonitis; IPF, idiopathic pulmonary fibrosis; IPAF, interstitial pneumonia with autoimmune features

During the study period, 216 patients diagnosed with interstitial lung disease underwent SLB at our hospital. A total of 28 patients were diagnosed with fibrotic HP by MDD at the time of SLB and 51 with IPF. Among the fibrotic HP patients, one had pulmonary alveolar proteinosis, and one had Sjögren’s syndrome. Among the IPF patients, one met the diagnosis criteria of interstitial pneumonia with autoimmune features. One patient each with IPF and fibrotic HP received steroids prior to SLB. One patient with IPF developed rheumatoid arthritis after biopsy. Excluding cases that met the exclusion criteria, 23 cases of fibrotic HP and 48 cases of IPF were considered in this study (Fig. 3). The ATS/JRS/ALAT Clinical Practice Guideline of the diagnosis of HP was published in 2020 [1], but the diagnosis in this study was made before this guideline was reported. The fibrotic HP diagnosis was again reviewed according to this guideline, and all cases of fibrotic HP diagnosed by MDD were found to meet the criteria for definite or high-confidence HP.

Table 1 presents the characteristic of the patients with fibrotic HP and IPF at the MDD. The serum Krebs von den Lungen-6 and surfactant protein-D levels were higher in fibrotic HP than in IPF patients. The percent predicted forced vital capacity (FVC), percent predicted forced expiratory volume in one second (FEV_1_), FEV_1_/FVC ratio, and percent predicted diffusion lung capacity for carbon monoxide (DL_CO_) at diagnosis were not significantly different between the two groups. The lymphocyte ratio and CD4/8 ratio in BAL were significantly higher among fibrotic HP patients.

**Table 1:** Patient characteristics

	Fibrotic HP n = 23	IPF n = 48	*P*-value
Age, years	68 (56–76)	67 (49–77)	0.362
Gender (male/female), n	15/8	35/13	0.506
Smoking status (ex/never)	12/11	32/16	0.239
WBC, μL	6250 (2530–10,300)	6365 (3400–13,500)	0.531
LDH, IU/L	232 (149–305)	211 (139–360)	0.428
CRP, mg/dL	0.11 (0.01–1.50)	0.14 (0.01–1.79)	0.526
KL-6, U/mL	1772 (578–8437)	834 (286–4666)	< 0.001
SP-D, pg/mL	393 (84–1111)	222 (45–897)	0.049
Pulmonary function			
FVC % pred	87.1 (63.8–125.5)	85.8 (44.9–137.1)	0.461
FEV_1_/FVC ratio	78.0 (65.4–96.6)	78.3 (59.5–100.0)	0.606
FEV_1_% pred	87.9 (67.0–115.4)	83.5 (52.1–129.9)	0.363
DL_CO_ % pred	78.5 (49.9–125.1)	76.5 (29.1–123.5)	0.588
BAL fluid^a^			
Total cell count, × 10^4^/μL	26.9 (2.6–69.1)	24.3 (0.2–62.1)	0.750
Macrophages, %	40.5 (4.0–91.0)	83.0 (17.0–96.0)	< 0.001
Lymphocytes, %	55.2 (7.5–95.0)	11.3 (1.6–70.0)	< 0.001
Neutrophils, %	1.4 (0–10.0)	2.0 (0–39.0)	0.425
Eosinophils, %	0.8 (0–6.0)	2.0 (0–18.0)	0.060
CD4/8 ratio	3.9 (0.2–9.8)	1.7 (0–8.4)	0.005

Data are expressed as median (range) or numbers of patients

HP, hypersensitivity pneumonitis; IPF, idiopathic pulmonary fibrosis; WBC, white blood cell; LDH, lactate dehydrogenase; CRP, C-reactive protein; KL-6, Krebs von den Lungen-6; SP-D, surfactant protein-D; FVC, forced vital capacity; FEV_1_, forced expiratory volume in one second; DL_CO_, diffusion lung capacity for carbon monoxide; BAL, bronchoalveolar lavage

^a^for BALF, n = 20 (fibrotic HP), n = 39 (IPF)

### HRCT assessment

Interobserver agreement across the two radiologists in the HRCT findings other than pleuroparenchymal fibroelastosis was fair to good. The kappa coefficient of hexagonal pattern was 0.44, indicating moderate agreement (Table 2). Extensive GGO, centrilobular nodules, and hexagonal pattern were more frequently observed in fibrotic HP than in IPF. A significant difference in axial distribution was also found between fibrotic HP and IPF.

**Table 2:** HRCT findings

	Fibrotic HP n = 23	IPF n = 48	*P*-value	Interobserver agreement	
				κ	95%CI
Distribution (zonal), n			0.611	0.22	− 0.02−0.47
Upper/middle predominant	0	1 (2.1%)			
Lower predominant	19 (82.6%)	43 (89.6%)			
Diffuse	4 (17.4%)	4 (8.3%)			
Distribution (axial), n			0.001	0.36	0.16–0.55
Peribronchovascular	2 (8.7%)	1 (2.1%)			
Peripheral	12 (52.2%)	44 (91.7%)			
Peripheral with subpleural sparing	4 (17.4%)	2 (4.2%)			
Diffuse	5 (21.7%)	1 (2.1%)			
Extensive GGO, n	19 (82.6%)	19 (39.6%)	0.001	0.38	0.16–0.59
Centrilobular nodules, n	20 (87.0%)	27 (56.3%)	0.010	0.27	0.07–0.47
Mosaic attenuation, n	5 (21.7%)	3 (6.3%)	0.102	0.21	− 0.12–0.55
Air trapping, n^a^	15 (65.2%)	25 (52.1%)	0.623	0.46	0.26–0.67
Three-density pattern, n	0	0	-		
Reticulation, n	23 (100%)	48 (100%)	-		
Honeycombing, n	3 (13.0%)	14 (29.2%)	0.136	0.52	0.24–0.79
Traction bronchiectasis, n	23 (100%)	48 (100%)	-		
Consolidation, n	1 (4.3%)	7 (14.6%)	0.261	0.48	0.11–0.85
Emphysema, n	7 (30.4%)	21 (43.8%)	0.283	0.59	0.39–0.79
PPFE, n	1 (4.3%)	10 (20.8%)	0.090	0.14	− 0.35−0.64
Cyst, n	8 (34.8%)	27 (56.3%)	0.090	0.69	0.52–0.86
Hexagonal pattern, n	16 (69.6%)	6 (12.5%)	< 0.001	0.44	0.19–0.69
HRCT patterns, n			0.000	0.37	0.22–0.53
UIP	0	4 (8.3%)			
Probable UIP	2 (8.7%)	26 (54.2%)			
Indeterminate for UIP	12 (52.2%)	14 (29.2%)			
Alternative diagnosis	9 (39.1%)	4 (8.3%)			

Data are expressed as numbers of patients

HP, hypersensitivity pneumonitis; IPF, idiopathic pulmonary fibrosis; GGO, ground-glass opacity; PPFE, pleuroparenchymal fibroelastosis; HRCT, high-resolution computed tomography; UIP, usual interstitial pneumonia

^a^One patient of IPF had no expiratory CT available

Mosaic attenuation, air trapping, or three-density pattern were reported as findings suggestive of fibrotic HP [1, 2, 6, 7], but no significant difference was observed in this study (Table 2). The logistic regression for predicting fibrotic HP is presented in Table 3. In the univariate logistic regression, the presence of extensive GGO, centrilobular nodules, and hexagonal pattern was associated with increased odds ratio of fibrotic HP. Extensive GGO and hexagonal pattern were the remaining variables in multivariate logistic regression. The presence of hexagonal pattern was highly specific (87.5%) for diagnosing fibrotic HP. The sensitivity for fibrotic HP diagnosis when hexagonal pattern was present was 69.6%. The positive predictive value of hexagonal pattern was 72.7% for fibrotic HP diagnosis.

**Table 3:** Logistic regression predicting fibrotic HP

	Odds ratio	95% CI	*P*-value
**Univariate logistic regression**			
Distribution (zonal)			
Upper/middle	0.00	0.00–Inf	0.991
Lower	0.44	0.10–1.96	0.282
Diffuse	Reference		
Distribution (axial)			
Peripheral	0.05	0.01–0.51	0.011
Peripheral with subpleural sparing	0.40	0.03–6.18	0.512
Peribronchovascular	0.40	0.02–10.00	0.577
Diffuse	Reference		
Extensive GGO	7.25	2.13–24.65	0.002
Centrilobular nodules	5.19	1.36–19.82	0.016
Mosaic attenuation	4.17	0.90–19.28	0.068
Air trapping	1.65	0.59–4.63	0.341
Honeycombing	0.36	0.09–1.43	0.147
Consolidation	0.27	0.03–2.31	0.229
Emphysema	0.56	0.20–1.62	0.285
PPFE	0.17	0.02–1.44	0.105
Cyst	0.42	0.15–1.16	0.094
Hexagonal pattern	16.00	4.66–54.91	< 0.001
**Multivariate logistic regression**			
Extensive GGO	4.86	1.20–19.70	0.027
Centrilobular nodules	1.45	0.30–7.08	0.647
Hexagonal pattern	11.00	2.70–44.70	0.001

HP, hypersensitivity pneumonitis; GGO, ground-glass opacity; PPFE, pleuroparenchymal fibroelastosis

## Discussion

This study has demonstrated that hexagonal pattern can be a useful finding for differentiating fibrotic HP from IPF.

HP is a diffuse interstitial pneumonia caused by immune response to inhaled antigen. Inhaled substances are usually most likely deposited at the level of the respiratory bronchioles, and small particles of 2.5 μm or less reach the alveolar region via diffusion, are phagocytosed by alveolar macrophages, and then enter the lymphatics. The distribution of lymphatic vessels in the lungs is largely divided into two routes. One lymphatic flow follows the bronchovascular bundles to the hilum in the inner layers. The other route starts at the perivenular area in the secondary lobule, runs through the interlobular septa or subpleural lymphatic vessels, and ends at the hilum [15]. In experiments using inhaled antigens, the most frequent site of granuloma formation in response to antigens is from the respiratory bronchioles to the alveolar ducts, as has been reported [16]. The deposition of inhaled substances into the respiratory bronchial habit is thought to be involved in the formation of lobular central lesions, whereas deposition by lymphatic flow is thought to be involved in the fibrosis of the lobular margins, i.e., subpleural and interlobular septal predominance.

Centrilobular nodules, extensive GGO, mosaic attenuation, air trapping, diffuse axial distribution, and upper or mid-lung predominance have been reported as useful HRCT findings for fibrotic HP diagnosis in previous reports [3, 4, 6, 8]. However, these studies differed in patient backgrounds or diagnostic methods, including with or without lung biopsy: some studies included nonfibrotic HP and fibrotic HP and other studies included other ILDs and IPF as control diseases.

The study comparing IPF with bird-related chronic HP that had histological UIP pattern reported that upper or mid-lung predominance and profuse micronodules were reported as key findings in chronic HP diagnosis [8]. The usefulness of the three-density pattern for differentiating fibrotic HP from IPF was reported, but in this study, not all patients underwent SLB [7]. The present study did not include cases with three-density pattern, and no significant difference in the presence of mosaic attenuation was observed between the two groups. This may be because this study included only those cases that required SLB as clinical information and radiological findings did not lead to the diagnosis. This suggests that the hexagonal pattern may be useful for differentiating fibrotic HP from IPF, even without a three-density pattern.

Fibrosis of HP manifesting as irregular reticulation is often visible on HRCT, which appears as thickened interlobular septa [1, 17, 18]. This finding usually correlates with the presence of fibrosis predominantly affecting the periphery of acini and the secondary lobule rather than the septa themselves. The study about HP in North India has reported that septal thickening was observed in nearly 30% of patients [19]. However, patients with IPF or other ILD with UIP pattern also often have irregular interlobular septal thickening [17].

This study proposed that not only the presence of interlobular septal thickening just below the pleura but also the extension of interlobular septal thickening to the inner layers, exhibiting a tortoiseshell-like pattern (hexagonal pattern), may be more characteristics of fibrotic HP than IPF.

In the case presented in Fig. 2, the area showing a hexagonal pattern was biopsied, and the hexagonal pattern in the HRCT was thought to correspond to perilobular fibrosis. However, in this study, SLB was not performed at selected sites with hexagonal pattern; thus, the HRCT findings and pathology in all patients enrolled have not been compared in this study. Since hexagonal pattern is a shadow that extends to the inner layers, in some cases, the shadow is found in the inner layers beyond the area that can be sampled by SLB.

Recently, the utility of TBLC has been reported in the diagnosis of diffuse lung disease [20–23]. Cryoprobe-retrieved specimens are larger than those of transbronchial forceps biopsies and less crush. TBLC tends to sample more proximal portion of the lung apart from the pleura compared with SLB. In the future, TBLC and SLB may be useful for comparing the imaging and pathology of the cases with hexagonal pattern in HRCT.

This study has several limitations. First, this was a single-center retrospective study, which may be subjected to various biases. External validation studies are warranted in the future. Second, this study included only patients who underwent SLB. Fibrotic HP and IPF, which can be diagnosed by clinical information, HRCT findings, and histological findings by transbronchial forceps biopsies or TBLC, were not included. However, the problem in clinical practice is the differentiation between IPF and fibrotic HP, which requires SLB. It is noteworthy that in this study, the hexagonal pattern was useful for differentiating between the two diseases, even in cases that the diagnosis could not be made based on clinical and imaging findings. Future studies including a cohort comprising of patients with fibrotic HP diagnosed without SLB should be conducted. Third, the interobserver agreement for hexagonal pattern was not high. However, the problem is not limited to hexagonal patterns only, but it is also seen with findings such as GGO and centrilobular nodules, which have been reported to be characteristics of fibrotic HP. Previous studies on HRCT findings in hypersensitivity pneumonitis have also shown only fair to moderate agreement among radiologists for these finding at rates comparable to this study [6, 8]. In this study, the interobserver agreement for hexagonal pattern was higher than that of GGO and centrilobular nodules. Therefore, we believe that the interobserver agreement in this study is not abnormally low, and it is simply necessary to examine more cases in the future. It is thought that a more accurate diagnosis of fibrotic HP can be made by combining multiple findings suggestive of fibrotic HP, rather than a single finding, and we hope that hexagonal pattern will be one of them. Finally, we did not examine whether the hexagonal pattern is useful in differentiating fibrotic HP from diffuse lung diseases other than IPF. In the future, we are planning to compare the HRCT findings of fibrotic HP with other diffuse lung diseases.

## Conclusion

Hexagonal pattern is a useful finding for differentiating fibrotic HP from IPF. Further external validation studies should be conducted to evaluate the utility of hexagonal pattern for diagnosing fibrotic HP.

## Data Availability

The dataset supporting the conclusions of this article is presented within the article. The detailed clinical data is not available because of patients’ confidentiality.

## Abbreviations

HRCT: High-resolution computed tomography
HP: Hypersensitivity pneumonitis
IPF: Idiopathic pulmonary fibrosis
MDD: Multidisciplinary discussion
BAL: Bronchoalveolar lavage
TBLC: Transbronchial lung cryobiopsy
SLB: Surgical lung biopsy
GGO: Ground-glass opacity
UIP: Usual interstitial pneumonia
